# Impact of Protein Corona on the Biological Identity of Nanomedicine: Understanding the Fate of Nanomaterials in the Biological Milieu

**DOI:** 10.3390/biomedicines9101496

**Published:** 2021-10-19

**Authors:** Md Habban Akhter, Habibullah Khalilullah, Manish Gupta, Mohamed A. Alfaleh, Nabil A. Alhakamy, Yassine Riadi, Shadab Md

**Affiliations:** 1 School of Pharmaceutical and Population Health Informatics (SoPPHI), DIT University, Dehradun 248009, India; 2 Department of Pharmaceutical Chemistry and Pharmacognosy, Unaizah College of Pharmacy, Qassim University, Unaizah 51911, Saudi Arabia; h.abdulaziz@qu.edu.sa; 3 Department of Pharmaceutical Sciences, School of Health Sciences, University of Petroleum and Energy Studies (UPES), Dehradun 248007, India; manish.gupta@ddn.upes.ac.in; 4 Department of Pharmaceutics, Faculty of Pharmacy, King Abdulaziz University, Jeddah 21589, Saudi Arabia; maalfaleh@kau.edu.sa (M.A.A.); nalhakamy@kau.edu.sa (N.A.A.); 5 King Fahd Medical Research Center, Vaccines and Immunotherapy Unit, King Abdulaziz University, Jeddah 21589, Saudi Arabia; 6 Mohamed Saeed Tamer Chair for Pharmaceutical Industries, King Abdulaziz University, Jeddah 21589, Saudi Arabia; 7 Department of Pharmaceutical Chemistry, College of Pharmacy, Prince Sattam Bin Abdulaziz University, Al-Kharj 11942, Saudi Arabia; y.riadi@psau.edu.sa

**Keywords:** nanoparticle, protein corona, biological entity, drug targeting, cellular uptake

## Abstract

Nanoparticles (NPs) in contact with a biological medium are rapidly comprehended by a number of protein molecules resulting in the formation of an NP–protein complex called protein corona (PC). The cell sees the protein-coated NPs as the synthetic identity is masked by protein surfacing. The PC formation ultimately has a substantial impact on various biological processes including drug release, drug targeting, cell recognition, biodistribution, cellular uptake, and therapeutic efficacy. Further, the composition of PC is largely influenced by the physico-chemical properties of NPs viz. the size, shape, surface charge, and surface chemistry in the biological milieu. However, the change in the biological responses of the new substrate depends on the quantity of protein access by the NPs. The PC-layered NPs act as new biological entities and are recognized as different targeting agents for the receptor-mediated ingress of therapeutics in the biological cells. The corona-enveloped NPs have both pros and cons in the biological system. The review provides a brief insight into the impact of biomolecules on nanomaterials carrying cargos and their ultimate fate in the biological milieu.

## 1. Introduction

**Figure 1:**
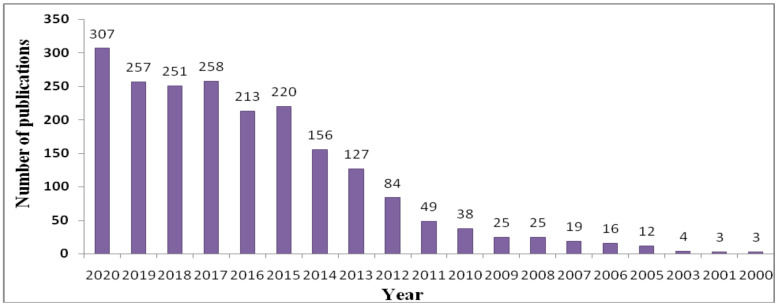
Timeline of entries in PubMed matching the search criteria “nanoparticles” and “corona” from 2010 to 2020.

The database access on PubMed makes it easy to judge a relevant scientific theme of important concern. As exemplified in Figure 1, a timeline search on PubMed with the title, “nanoparticle and corona” disclosed > 2000 publications from the years 2000 to 2020, and this number has increased eight-fold in the last decade. The surface coating of the NPs with PC in a living organism is indeed a prominent unresolved topic of discussionfrom a scientific and economic point of view. For decades there has been a steadily growing interest in the analysis of nanomaterials in biological fluid to trace the true molecular structure as expected during the preparation. This is valuable for nanotechnology and for biomedical and theranostic application. There is a wide gap between the drug discovery process and the biological identity of nanomaterials in the biological milieu. It has been identified that the surface of NPs alters due to the adsorption of protein, lipids, and biomolecules post-incubation in biological fluid. The protein-masked nanomaterials behave as new biological entities inferred as protein corona [1,2]. The formation of corona is a dynamic phenomenon governed by a lowering of the surface free energy in which various biological substrates compete for the same binding site onto the nanocarrier surface [3]. The protein adsorption on the surface of nanomaterials is considerably affected by their type, geometry, and conformation in biological fluid [4]. A lack of understanding associated with the in vivo performance of nanocarriers related to their physico-chemical characteristics, nano–biointeractions, blood circulation time, and corona formation could be rational explanations for the minimal translation of nanomedicines into clinical practice. The binding of proteins to the nano-surface is a complex, unpredictable process, affecting the fate of NPs related to biological and toxicological responses [5,6].

Numerous attempts have been made in the past to chemically modify the nanosurface to combat protein adsorption on to them. For instance, the functionalization of the NPs’ surface with the polyethylene glycol (PEG) chain through PEGylation was designed to make it biocompatible and to allow a specific interaction with the cells and tissues of the biological compartment. However, the PEG-modified surface of the NPs retained colloidal stability in physiological fluid with reduced protein adsorption, less opsonization by RES, improved circulation, and biodistribution. However, it is difficult to combat the bio-corona formation on NPs [7,8]. In particular, Petry et al. (2019) performed a study to prevent the destabilization of colloidal silica nanoparticles by adding a depletion polymer of Pluronic-F127 and PEG of different molecular weights. The protein band intensity indicated that PCs of varying molecular weights (MW< 17 kDa to >135 kDa) were accessed by a silica particle surface. It was found that the used polymer was adsorbed on surface of a silica particle and BSA maintained the electrosteric stability of colloidal dispersion, whereas PEG and PF-127 governed the depletion force in between the particle. Despite the interaction between the polymer and silica NPs, the protein coating to the NPs surface was not prevented and had the least possible influence on PC formation when they incubated with blood plasma. Thus, the serum protein had a greater effect on the corona profile compared with the polymers PEG and PF-127 [9].

Silver NPs (AgNPs) have been established and have a wide application in biotechnology and biomedical sciences. Despite their use as biocides, they are also used in tumor therapy, imaging, and as sensing agents. In a similar attempt by Batista et al., incubated polymer-layered AgNPs and self-generated a model protein with the intention to evolvePC. The polymers used for coating as stabilizing agents were polyethyleneimine (PEI), polyvinylpyrrolidone (PVP) and poly(2-vinyl pyridine)-b-poly(ethylene oxide) (PEO-b-P2VP). The PEO-b-P2VP and PVP-capped AgNPs were found to be inert to the adsorption of the self-generated protein model. In contrast, PEI-capped AgNPs interacted prominently with the BSA protein, which was probably due to hydrogen bonding and the Van der Waals force of interaction. Moreover, the same colloidal particle of silver established stability in the lysozyme and immunoglobulin G (IgG) environments [10].

The PEGylation nevertheless remains the gold standard for the stealth modification of nanocarriers in drug delivery. The challenge ahead to combat the complete inhibition of the PC coating to NPs remains critical. In relation to this, Wang and collaborators tried to minimize the PC covering on gold NPs by improving the stealth property of the PEG. The PEG terminal was conjugated with the α-glutamyl group and then polymeric micelles with α-glutamyl-terminated PEG shells were prepared. Thereafter, the polymeric micelles were incubated in fetal calf serum. The results demonstrated that a little change in the micelles size with the α-glutamyl group PEG shell was observed compared to a marked change in the size of same micelles without this group. Further, the micelles with the α-glutamyl group PEG shell evidently showed low protein access to the NPs and a long circulation time compared to the micelles without the α-glutamyl group PEG shell. Conclusively, the improved stealth effect of the micelles with the α-glutamyl group PEG shell was likely due to the zwitterionic characteristics of this group [11]. To identify the degree of association between the protein and the NPs, extensive studies are required to address the precise nanocarrier features, the covering of hard or soft protein corona, and nano–biointeraction [12,13,14]. Compared to a decade ago, the publications covering the identity of NPs in biological systems and the concept of corona have increased 15-fold. However, the assessment of proteins over nanocarriers in biological mediums is difficult to carry out. Moreover, PC formation is conditionally beneficial in the biological system, as the living cells exposed to the bare NPs may pose a threat to them compared to the corona-layered NPs until the bare NPs are sequestered via the macrophagic system [15].

The investigation of the new identity of nanomaterials in biological fluid due to PC formation is relatively advanced and represents a novel area of research. Indeed, the critical challenge is to identify the actual composition, size, and surface chemistry of the corona particles as they continuously change their surface morphology over time and adapt to the composition of the biological environment. In-depth studies are required to better understand the biological modification of nanocarriers, their composition, and the structural integrity of nanocarriers due to corona particles [16].

## 2. Types of Coronas and the Biological Identity of NPs

**Figure 2:**
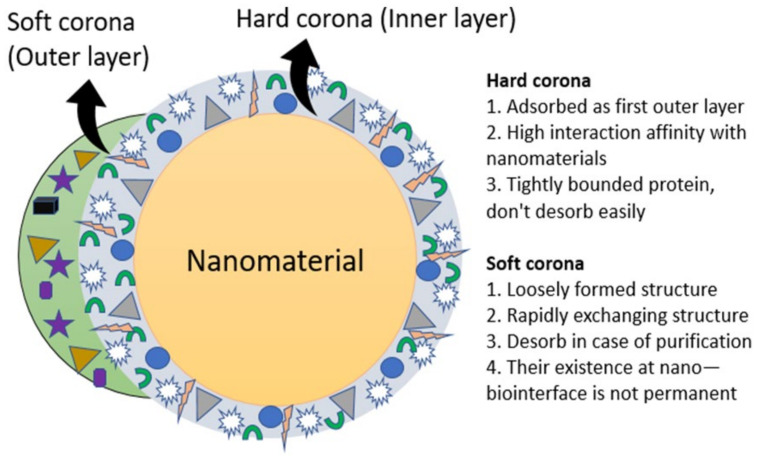
A hard versus soft protein corona model on the surface of nanomaterials in the biological milieu. Hard corona is analytically accessible by the NP–protein complex which is adsorbed primarily when NPs are in contact with a biological medium. It is tightly bound due to a high degree of affinity and isdesorbed easily. In contrast, soft corona is loosely bound and rapidly exchangesbiomolecules. The term protein corona/biomolecule corona is exchangeable with hard corona or analytically accessible corona.

When the nanomaterials enter into systemic circulation, they are exposed to various biological components such as blood, cells, lymph, plasma protein, and other biomolecules. During circulation in the blood, nanosize materials positively interact with these components over time. The adsorption of biomolecules to the surface of the nanomaterials leads to the generation of PC. Further, apprehending the protein corona–NPs association, their exchange and quantification and protein affinity for NPs has been well established by Cedervall and associates. PC is a complex structure that is generally 20–30 nm thick and has both a hard and soft consistency. The hard corona complex covering the nanocarrier is stable with a long retention time compared to the soft corona on the same nanocarrier [17]. The hard corona is formed with a high interaction capability with NPs, resulting in a nearly permanent structure which has an exchange time that is higher than the cellular intake of the particle [12,18]. The protein that binds loosely to the corona directly on the surface of the NPs or that weakly binds to the hard corona surface of the same NPs can easily exchange in a biological medium due to the weaker protein–protein interaction formed between these layers that together forms a structure called a soft corona. It is generally accepted that hard corona formation takes place primarily with NPs following a low affinity protein interaction between them. The hard versus soft corona formation on the NPs surface is illustrated in Figure 2. It is also assumed that the formation of hard and soft coronas depends upon the affinity or the competing ability of proteins towards the binding site on the surface of NPs [19].

**Figure 3:**
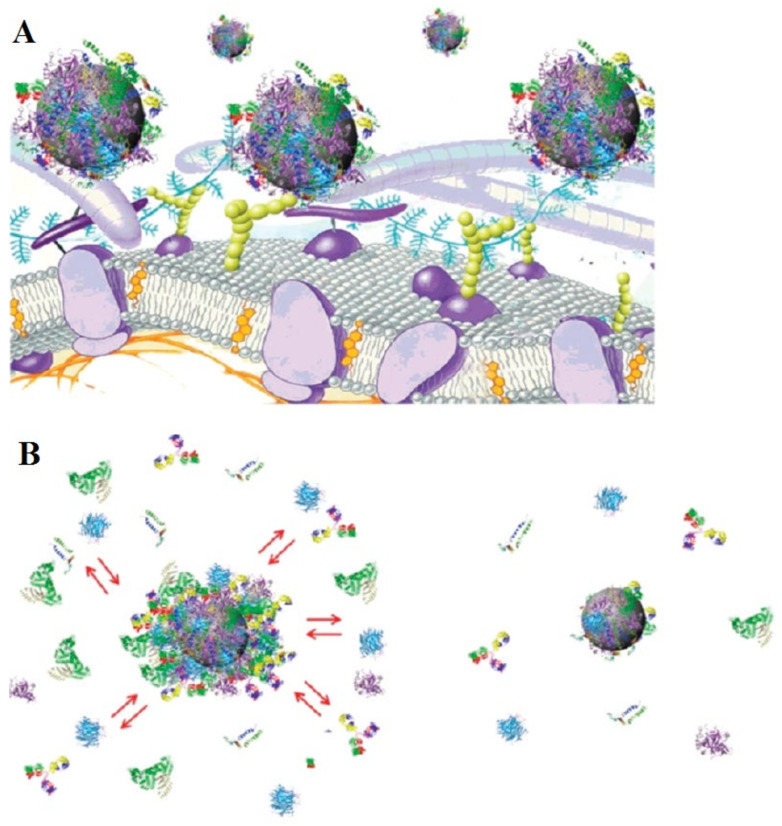
Illustrative diagram showing the interaction/exchange script and the probable NP–protein complex structure. (**A**) Representative drawing shows the possible exchange/interaction scenarios at the bio-nanointerface at the cellular level. (**B**) Representative drawingshows the structure of the NP–protein complexes in plasma affirming the outer weakly interacting layer of protein (left, full red arrows) and the hard slow exchanging corona of proteins (right). Adapted, with permission, from [19].

Walkey and associates proposed that hard corona is considerably more important than soft corona in predicting the fate of nanomaterial in biological fluid [20]. Therefore, understanding the critical role of hard and soft corona formation to the NP surface and the biological response of PC in vivo is important in the rational design of NPs. The protein interaction/exchange script and the probable NP–protein complex structure are shown in Figure 3A,B. One study reported on the nature of corona on the surface of iron oxide NPs by incubation in fetal bovine serum (FBS) and found that the protein layers around the NPs were anti-thrombin, and α-antiproteinase, a soft corona [21]. Further, the nature of hard corona on magnetic NPs and their biological behavior were investigated by Bonvin and colleagues. They separated proteins from the surface of the NPs using a multi-step centrifugation technique for one-domain magnetic NPs and compared this with the previously defined magnetic separation technique. The multi-step centrifugation separation technique was employed while incubating iron oxide NPs in human blood and lymph serum in various dilutions. The composition of hard protein corona obtained showed the reproducibility of the multi-step centrifugation technique. Later, it was compared with existing techniques for obtaining the separation of magnetic nanoparticles. Accordingly, the study on hard corona established limits for validity on both the techniques used. Surprisingly, the hard corona obtained in these two techniques was quite different [22].

## 3. Separation Technique of Protein Corona

The isolation of the corona complex system from the biological matrix is an important exercise that needs attention and it can be critically investigated during the separation process. NPs encased by PC can be analyzed by different methods using various separation techniques. The separation of corona-bound particles from the surrounding medium includes important processes such as centrifugation, chromatography, and magnetic separation. The centrifugation-based separation technique is intended to capture a larger portion of hard corona proteins for identification and to examine in vivo behavior. Pelletization is one of the centrifugation techniques used for the separation of protein corona from the nanocarrier complex from the surrounding matrix. In the first instance of the centrifugation process, the loosely bound proteins are not completely removed from the surface of the NPs; therefore, washing in a buffer solution is repeatedly required to ensure the same result. The speed and time of centrifugation is notably optimized to ensure the complete separation of the protein corona–NPs complex from the surrounding matrix while preventing aggregates or pellets at the end point [23,24]. The microscopic examination of the centrifuged material using TEM has revealed protein corona and a poorly defined network of loosely bound proteins probably due to the bulk capture of protein within these networks. Further, asymmetric flow field-flow fractionation and surface plasmon resonance (SPR) coupled with mass spectroscopy have been implemented to PEGylated NPs to recognize the weak association of protein corona in the stealth system. Later, it was identified that the soft corona protein was a key component of the stealth system and its biological identity [22,25,26,27].

Another consideration in the separation of the corona system is the thorough washing that needs to be repeated many times during the centrifugation process to separate the particular NP–corona complex within the protein-rich medium [23]. Konduru and coworkers reported that the true density of NPs cannot be exactly measured from their density due to the agglomeration and the medium effect in the biological system. A suitable control must be subjected to evaluate NPs in the supernatant, and protein aggregates precipitated in the pellet [28]. The alternate technique is the magnetic separation of NPs from PC in biological fluid which provides a rapid and easier approach. The magnetic separation of corona from the NP complex is similar to the centrifugation process; the only additional step is the analysis of the surface adsorbed protein via liquid chromatography–mass spectrometry (LC-MS) [22]. Chromatographic separation is another approach, but it is less frequently used, possibly due to the cost burden and the fact that it is a time-consuming process and has a comparatively low turnout [23].

## 4. Impact on the Physico-Chemical Characteristics of NPs

The physico-chemical attributes such as particle size, shape, surface area and surface charge significantly affect the protein adsorption and thus modulate the surface characteristics of the NPs in the biological system. The formation of PC strongly changes the fate of NPs and gives them a new biological identity in vivo. To investigate the impact of particle size, Xu et al. developed lipid membrane-enveloped hybrid NPs for specific lipid-receptor oriented targeting. The authors claimed the hybrid NPs were comparable to artificial viral NPs (AVNs) which are comprised of a gold core (AuNPs) layered with bi-layer phospholipid and a surface functionalized with ganglioside GM3 (ligand) for active targeting to the CD169-expressing antigen presenting cells. The size of the developed formulation containing different % of 1, 2-dioleoyl-sn-glycero-3-phospho-L-serine (DOPS) over the functional surface reported a diameter of 35 or 80 nm. The larger particles with a low concentration of PC were indicated by a small rise in the particle diameter measured by a particle size analyzer. Further, in the case of a greater percentage of DOPS conjugation to the AVN surface, more bound proteins were detected. Thus, bigger particles with a low percentage of DOPS showed a higher stability in serum plasma. As a result, the high layering of PC to artificial viral NPs led to a reasonable lowering in the targeting potentiality and the cellular uptake of CD169-expressing antigen cells in vitro. Additionally, the membrane-wrapped NPs targeting the GM3 moiety offered a biomimetic platform for the rational design of corona-repellent properties [29].

Interestingly, García-Álvarez and associates demonstrated the impact of the shape of NPs on the PC in vivo. The various shapes of NPs i.e., gold NPs, nanorods, and nano-star configurations were incubated in animal blood and analyzed for the binding of the specific protein [30]. The analysis revealed that the majority of PC apportioned on both nanorods and nano-star NPs and the abundance of various shaped proteins made it clear that the shape of NPs could be decisive for PC composition. For instance, the nanorods had a double concentration of serum albumin than the nanorods corona. Further, to investigate the impact of size of NPs on the PC, different sizes of iron oxide particles (30, 200, and 400 nm) were incubated in human serum. It was found that 20% of the total corona was apportioned, indicating that the size of NPs is crucial in PC formation [31]. Bewersdorff et al. studied the impact of the shape and charge of the gold nanostructure on PC formation. They used four comparable size and shapes such as spheres, rods, stars, and cages of nanometer scale. These nanomaterials were charged with a heterofunctional linker. The LC-MS analysis revealed that the influence of shape was greater than the surface charge. The cage shaped gold NPs showed a low concentration of corona protein viz., albumin, vitronectin, and other complement proteins, which were probably due to high ligation and curvature areas over the flat surface which favor dysopsonization and a rapid clearance from the immune system [32].

The particle surface area and the abundance of macromolecules in the biological sample affected the PC formation. The ratio of biological fluid and the surface area of NPs have an impact on the PC formation on the surface of the NPs.

Lundqvist et al. illustrated the influence of particle size on formed corona around silica NPs of different sizes, 13 and 23 nm. The surface areas of both particles were the same for the 9.5 nm particles, i.e., 4-fold greater than the 76 nm particles. The corona detected for the 76 nm sample in the whole blood samples resembled apolipoprotein A-I with aprominent band, rather than the 9.5 nm sample. In serum, the detected corona was in a changeover from 9.5 to the 76 nm NPs i.e., the quantity of apolipoprotein A-I reduced with the increase in NP size due to the change in size or curvature of the NPs [33]. Besides the particle size, the PC detected around the NPs also depended upon parameters such as the composition of the biological medium, and the fraction of the particle surface area in direct contact with the biological medium.

If the surface area of the NPs is large i.e., if a large number of particles are present in a given area, a low affinity protein may bind and stabilize the interaction with the aggregates of NPs. The exposed surface area may also reflect the protein-compelled NP aggregation. The trapped protein in the complex can be analyzed after its release from the NP–protein corona complex although it is not a part of corona composition. The large surface area aggregates of the corona with diversified proteins have been explained in previous work [34,35].

**Figure 4:**
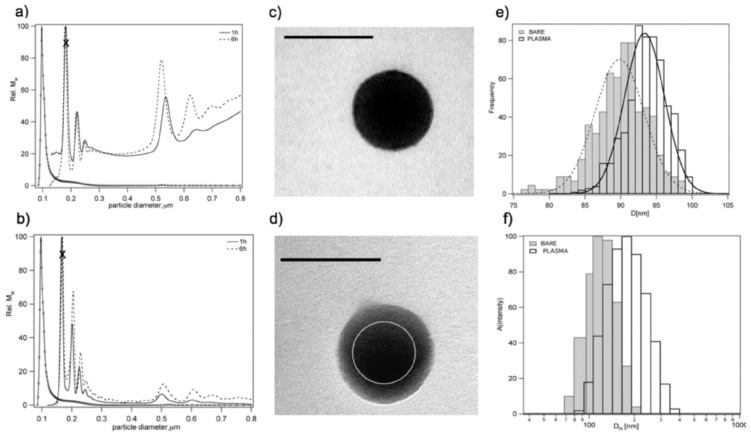
(**a**) Differential centrifugation sedimentation results (DCS) for 100 nm PSCOOH NP–protein complexes in plasma measured after 1 h (solid line) and 6 h (dotted line) of incubation. (**b**) DCS results for particle–corona complexes free from excess plasma 1 and 6 h after in PBS. Bare = 100 nm. PSCOOH NPs in PBS (open circles) for reference in both graphs. The marked peaks relate to the monomeric NP–protein complexes. (**c**) Transmission electron microscopy (TEM) picture of bare 100 nm PSCOOH nanoparticles. Bar = 100 nm. (**d**) TEM picture of the protein–particle complex (free from excess plasma) for 100 nm PSCOOH NPs. (**e**) Size distribution histogram by size analysis of various TEM images of particles. (**f**) Dynamic light scattering (DLS) intensity-weighted size distributionfor 100 nm PSCOOH NPs (bare) and 100 nm PSCOOH protein–particle complexes free from excess plasma (washed corona) in PBS. Adapted, with permission, from [19]. Copyright (2020) American Chemical Society.

Walczyk et al. conducted a study on several normal nanoparticles dispersed in blood plasma to investigate the formation and categorization of (hard or soft) PC assembly and their longevity. Herein, the authors used carboxylated polystyrene particles (PSCOOH), sulfonated polystyrene particles of sizes 100 and 200 nm, and silica particles of size 50 nm incubated in normal plasma. The results are shown in Figure 4a–f. In Figure 4a, it can be seenall the changes in the peaks during incubation were reproducible. Figure 4b shows that the protein complex monomer and the bigger protein–particle complexes were retained with unaltered sizes as in the plasma in situ. Figure 4c,d indicates that the particles werepolydisperse in nature, and that protein conformation is affected by the method of sample preparation. Moreover, the size of corona particle was observed to be 5 nm larger than the bare nanoparticle (Figure 4e). The hydrodynamic diameter of corona particles was found to increase by 50 nm compared to thebare nanoparticles (Figure 4f).

**Figure 5:**
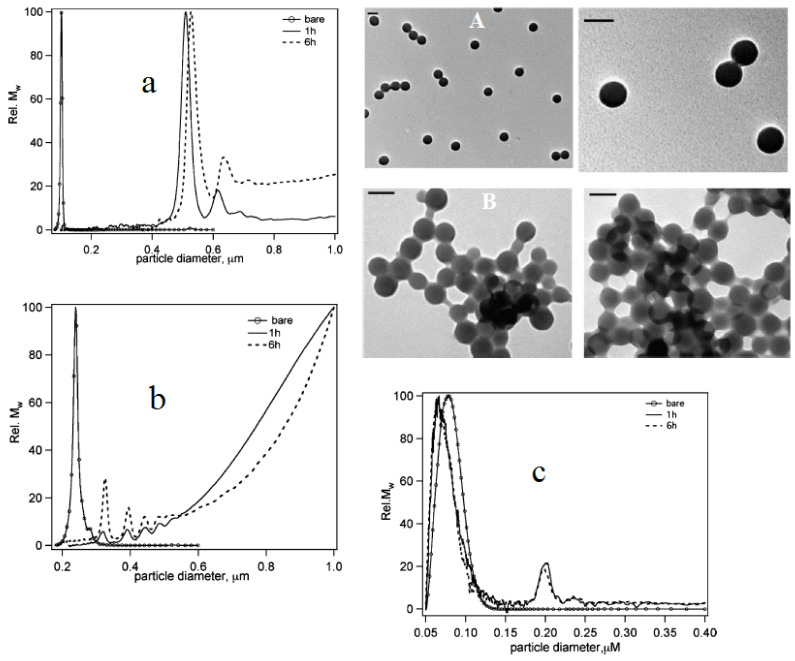
Time-resolved DCS experiments of 100 and 200 nm PSOSO_3_H and 50 nm SiO_2_ NP–protein complexes. (**a**) DCS of 100 nm PSOSO_3_H NP–protein complexes free from excess plasma (washed system) as a function of time. (**b**) DCS of 200 nm PSOSO_3_HNP–protein complexes free from excess plasma. (**c**) DCS for 50 nm SiO_2_ NP–protein complexes free from excess plasma. In all graphs, the bare NP results (open circles) are reported for reference. TEM images of bare 100 nm PSOSO_3_H NPs at different magnifications (**A**). TEM pictures of 100 nm PSOSO_3_H protein–NP complexes free from excess plasma (**B**). Bar = 100 nm. Adapted, with permission, from [19]. Copyright (2020) American Chemical Society.

The hard corona of sulphonated polystyrene (PSOSO_3_H, 100 nm, 200 nm) and SiO_2_ (50 nm) nanoparticles are shown in Figure 5a–c. For bare PSOSO_3_H particles of diameter 100 nm in PBS, the monomer was consistent with the TEM image (Figure 5A) but the particle monomer peak was absent in the plasma mixture and the plasma intrinsic peak grew rapidly. The large cluster formation associated with a larger amount of protein (NP–protein complex) was confirmed in 100 nm sulfonated polystyrene, as shown in the TEM images, compared to the carboxylated NPs, where only a thin protein layer and no monomer complexes were detected (Figure 5B) [19].

The exposed charge surface of NPs in the biological milieu is a prominent feature for the determination of PC and their type of formation. The composition of PC is affected by the presence of the surface charge of NPs and their interaction with biomolecules restrains the fate of protein adsorption kinetics. To elucidate the impact of the surface charge of NPs on corona formation, Lundqvist and coworkers used polystyrene NPs withpositive (+) amine and negative (−) carboxyl terminals incubated separately in human plasma and estimated the influence of the surface charge as a significant outcome [34]. The proteins susceptible to positive NPs were apolipoprotein F, mannose binding proteins, complement C1r, and the proteins susceptible to negatively charged NPs were majority of the Ig-gamma and Ig-kappa proteins.

In spite of the protein adsorption over the surface of NPs, the zeta potential remained unaltered in the range of −10 to −20 mV, indicating autonomous control over the physico-chemical properties. Furthermore, Alkilany and associates studied both cationic and anionic polyelectrolyte-surfaced gold nanorods and obtained the same zeta potential as above, −20 mV, after incubation in a biological medium with bovine serum albumin (BSA). Thus, the finding led to the conclusion that the protein adsorption on the surface of the NPs apart from physico-chemical features of NPs depends upon several other critical factors related to the biological milieu in vivo [36].

To further understand the impact of the surface charge of NPs, Almalik et al. prepared chitosan NPs (CS NPs), functional hyaluronic acid-coated chitosan (HA-CS) NPs, and alginate-coated chitosan NPs (Alg-CS) NPs using a previously optimized methodologyand studied them in a biological buffer and in serum to investigate the interactions between the NPs and the biomolecules. The CS NPs indicated a high positive zeta potential in the buffer as expected. The surface coating of CS with anionic HA led to a positive charge of the CS NPs, whereas the adsorption of polyanion alginate altered the charge from positive to negative in the CS NPs. Moreover, the alginate CS NPs showed more negative zeta potential and an increased diameter compared to the HA-coated CS NPs. The analysis of the association of CS NPs with the serum protein showed that the intense affinity with the protein and the formation of dense PC was due to the cationic charge. In contrast, the fact that the size distribution in HA-coated CS NPs demonstrated insignificant changes after incubation in serum may have been due to the lower adsorption of the protein due to the moderate negative charge densities [37,38,39].

The impact of the molecular weight of CS on the physico-chemical properties of NPs was investigated. The high molecular weight of chitosan had more porosity and a low cross-linked density, and this brought a large dimensional change in response to the change in osmotic pressure. The porosity in the chitosan nanoparticles in accordance with their molecular weight had a significant effect on the surface of HA around the NPs. It was evidently established that HA deeply penetrated into the abundant porous structure of high molecular CS NPs. On the other hand, densely packed cross-linked low molecular weight chitosan NPs experienced a layer of corona. An atomic force microscopic study investigated the occurrence of dry corona of a thickness of about 20 to 30 nm. The study revealed that molecular weight has a profound effect on how HA is portrayed to protein, which further suggests that the specific interactions with CD44 receptors have control over the release kinetics and cell uptake of nanoparticles [36].

## 5. Impact of PC on the Identity of NPs in the Biological Milieu

The rapid advances in the area of NPs and nanotechnology have revolutionized their application in engineering and the material and biomedical sciences. The information deficit and the lack of understanding of the phenomena occurring in the biological milieu at the nano–biointerface with NPs could be one of the causes for the limited success in clinical settings, although a few of them have entered into clinical use. The systemic exposure of NPs to a wide range of protein concentrations in the blood and interstitial fluids leads to the formation of a PC layer on the surface of the NPs, steadily modifying their identity which is referred to as a “biological identity” [40]. The concept of NP–protein interaction has been well established in the research domain [41,42,43] but the impact of this interaction on biological responses has been deliberately underestimated. The reason behind this could be due to the fact that protein adsorption around the surface of the NPs was long perceived to have a detrimental effect on the NPs, resulting in an unknown biological identity and untoward effects including recognition by the reticuloendothelial system and the immune system, and the removal from the blood circulation [44]. Taking this into account, NPs should be designed and developed with a modified surface technology that could resist the covering or adsorption of protein around the NPs’ environment. A productive insight favoring the study was the development of PEG, hydrophilic polymer-coated NPs which resulted in steric hindrance and foreshortened the surface adsorption of serum/plasma protein [45]. Dawson and associates revealed the NP–protein interaction when NPs approaching the biological media were surrounded by a layer of dynamic coating protein molecules i.e., PC [2,34]. Furthermore, Stauber et al. explained that PC is factually a protein in-build over NPs due to the interaction with protein. It is generated rapidly as a complex structure which grows quantitatively over time with concept of the Vroman effect (protein binding kinetics) [46]. This effect postulates that the process of biomolecule adsorption on the NPs’ surface is a time dependent process in which the highly abundant and predominantly adsorbed protein firstly accompanied by the less abundant protein with a high-binding affinity results in a complex phenomenon involving the kinetics of association–dissociation [47,48]. The structure and composition of PC depends upon the features of the NPs such as shape, size, composition, zeta potential, exposure time, and biological environment. The current findings also suggest that PC grows quantitatively with minute qualitative modification, which also occurs over time. With the evolution of new surface properties of NPs in the biological milieu irrespective of the original characteristics, the PC obtains a new identity of NPs that are largely accessible and have been perceived in the living organism [49]. The impacts of the physico-chemical characteristics of nanomaterials in PC formation in the biological milieu are shown in Table 1. A few examples of the nanomaterials that integrate with proteins forming corona in blood plasma/serum are expressed in Table 2.

## 6. Impact of the Flow Dynamics on Corona Formation In Vivo

To understand corona formation, large numbers of data are available on in vitro studies on the nano–biointeractions in the blood, plasma, and serum as well as in a culture medium. The data from in vitro studies recognized the diversity in the structural organization of the living organism [67]. Moreover, few studies have reported that corona formation on nanomaterials is difficult to identify in vivo owing to the trouble in capturing them in the biological system. Given the gaps in the knowledge related to the understanding of the precise nano–biointeractions in vivo, it is no wonder that few NPs have translated from bench to bedside. The dynamic nature of fluid flow (blood) in the human body may cause some stress on NPs and act a source of adsorption of biomolecules [51,52,53]. To enrich the understanding of the impact of fluid flow dynamics on PC formation in a biological medium, investigators explored the role of shear stress on the structural integrity and surface composition of PC. They choose lipid vesicles with different surface chemistries then exposed them to FBS in static and dynamic conditions, both provided by a peristaltic pump. The PC that evolved around un-PEGylated NPs under dynamic conditions recorded a high concentration of apolipoproteins APOA-II, while under static conditions, acute phase proteins and alpha-1-antitrypsin were traced. Surprisingly, in PEGylated NPs, a total of 217 proteins were detected, of which 50% of the proteins involved in the corona were incurred under dynamic conditions and some of the coronated complement protein was identified as C3 and C4 with opsonin activity. This finding is important in relevance to the in vivo condition because a complement protein is largely associated with the clearance of NPs from systemic circulation [68,69]. Braun and colleagues investigated the influence of the dynamic flow conditions on AuNPs that were 13 nm in size with a surface coating of PEG and tannic acid. The dynamic system was designed using a peristaltic pump at a velocity of 0.5 cm/sec. It was established that the PC composition influenced the dynamic flow in synthetic fluids and the alteration in the PC relied highly on the surface designed and the chemistry of the AuNPs [51]. Additionally, some reports evidently established that corona formation differs in dynamic and static flow environments and the molecular complexity of proteins in the dynamic flow is greater than in the static flow. Notably, the latest technological advances would be adjuvants inthe prediction of the fate of NPs in vivo regarding the biodistribution and the circulation time in the blood which includes the 3D cell culture and organs-on-a-chip devices as strategic models. These advance models could drastically reduce the experimentation in animals and may provide more realistic features of PC composition and a clear understanding of the implication of PC on biological responses [70].

## 7. Impact of Incubation Time, Temperature, Shear Stress, and the pH of the Media

Incubation time, pH, temperature, and shear stress can either be seen as having an individual impact or a combined impact on PC formation on the NPs’ surface. The impact of incubation time, temperature, shear stress and the pH of the media have been well studied in relation to the formation of the bio-molecular corona. It has been observed that protein adsorption on the surface of NPs commences within half a minute of incubation in the biological medium. To understand the combined influence of incubation time and temperature on corona formation, Weidner and coworkers studied magnetic iron oxide by incubating it in fetal calf serum at different temperatures for 20 min. The sodium dodecyl-sulfate polyacrylamide gel electrophoresis (SDS-PAGE) investigation disclosed that a higher quantity of protein was adsorbed at 25 °C and 37 °C. Further increasing the incubation temperature to 50 °C and 70 °C showed that the protein bound to the NPs’ surface was a denatured protein of molecular weight ranging between 25 and 75 kDa [54]. The colloidal AuNPs are perceived to be versatile nanoplatforms for biomedical use. Dobrovolskaia and associates investigated the role of the surface characteristics and the incubation time on the pattern of corona formation on colloidal PEGylated AuNPs. The PEGylated gold NPs of 30 nm in size were incubated with plasma at room temperature and 37 °C and it was reported that the total amount of protein that bound to the surface of the NPs decreased with an increase in time from 5 to 60 min, while the composition of corona remained unaltered. Further, the protein binding was governed by the level of the PEG coating [6].

**Figure 6:**
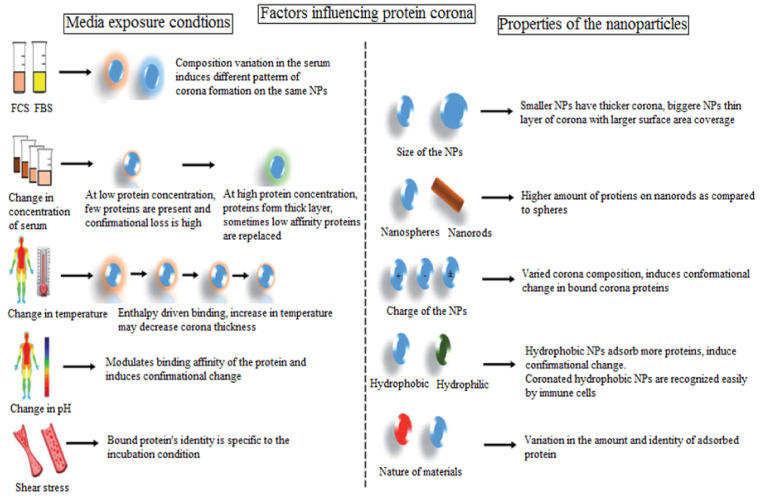
Influence of the properties of NPs and the incubation medium, temperature, and pH on PC formation. Permission under Creative Commons Attribution-Non Commercial 3.0 Unported License [72].

Furthermore, the impact of temperature on the protein composition over the NPs’ surface was investigated with different designs and surface charges by Mahmoudi and associates. The results suggested that temperature had an important role in the protein composition and the adsorbed protein and that caution was required in monitoring the quantitative studies at the nano–biointerface [3]. As illustrated in Figure 6, the external factors such astemperature and pH can alter the adsorption at the NPs surface. At a high temperature, the binding of BSA to the quantum dots was lowered [50]. The observation on copper and magnetic NPs also showed that temperature change had an important influence on the pattern, formation, and covering of PC, respectively [71]. The fluorescence correlation spectroscopy established that the binding feature of BSA to quantum dots (QDs) varied by changing pH from 6 to 9. At a lower pH, the binding affinity reduced, which could be attributed to a repulsive force, whereas at a higher pH, more binding to the QDs was observed, which was probably due to the loss of conformation change in the protein structure. This study further supported the same reaction inside the cells i.e., at a lysosomal and endosomal pH, the conformational change and biological activity of the PC altered [55].

The influence of blood flow in the body marked as shear stress set forth a change in PC formation on the surface of PEG-coatedliposomes. The mass spectral and DLS demonstrated a higher size of the NPs compared to particle size in vitro after incubation in FBS. A library of proteins was identified on the liposome surface under both dynamic and static conditions. The complement protein was abundantly examined under dynamic conditions, while albumin, alpha-2-HS-glycoprotein, and transferrin were reported under static incubation. The influence of the overall factors is summarized in Figure 6 which demonstrates the substantial impact on PC formation [72].

## 8. Impact on Drug Release Kinetics

In designing targeted release drug delivery systems or any other drug delivery system, drug release is a critical parameter in vitro that is essentially estimated before proceeding to the next parameter. The administration of NPs is irrespective of the route ofcontact with the biological fluid surrounded a library of blood protein depending on their physico-chemical properties or biological environment. The formation of bio-molecular corona generally resulted in a decrease in the drug release or in some cases increased the drug release from the nanocarrier [73]. The formation of PC layers around the NPs’ surface reduced the effective burst release [58]. The release profile from albumin-bound paclitaxel commercialized as Abraxane^®^ altered due to shield PC. Moreover, the PCs significantly diminished the drug release behavior from SPION, and the camptothecin release from silica NPs indicated a slower release due to protein corona [59].

Sebak et al. developed poly-lactic co-glycolic acid (PLGA) NPs and characterized them in vitro to assess their physico-chemical properties. The PC decoration of these NPs was analyzed both qualitatively and quantitatively using Western blot and Bradford assays, respectively. The influence of PC on the drug release behavior from NPs and their intracellular uptake into melanoma cells (B16F10) has been established. Moreover, they prepared two compositions of NPs namely, bare polymeric PLGA-NPs and peptide ligated hybrid NPs (cRGDyk peptide) and incubated them in plasma to assess the PC makeup of these NPs. The in vitro drug release from the NPs significantly depended on the PC make up as well as the concentration of the serum proteins in the drug release medium. The drug release was higher in the presence of proteins in cRGDyk peptide PLGA-NPs, while a reduced release was observed in the non-conjugated NPs. Further, intracellular uptake of NPs in melanoma cells laid down the important role of serum protein and cellular protein accumulation in NPs. For instance, vitronectin protein in serum is a good sign of the positive intracellular uptake of cargoes [60].

## 9. Drug Targeting and Cellular Uptake in the Biological Milieu

**Figure 7:**
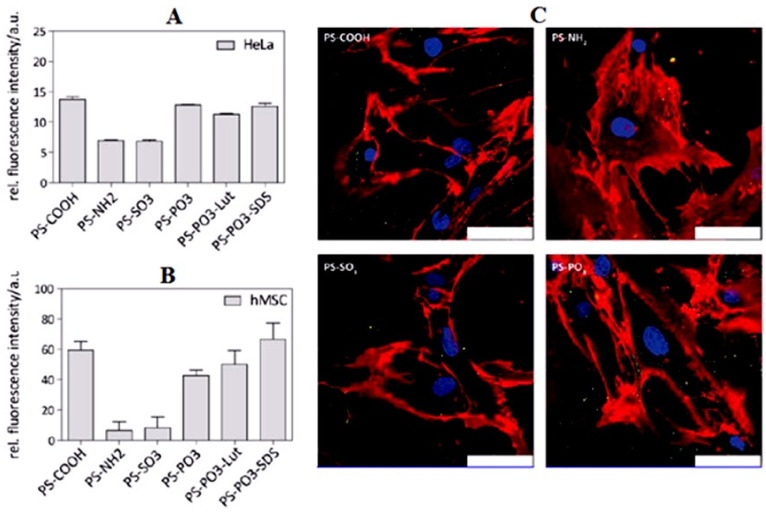
Biological uptakes of carboxy (PS-COOH), amino (PSNH_2_), sulfonate (PS-SO_3_), or phosphonate (PS-PO_3_) functionalized polystyrene nanoparticles in human cells. Quantitative estimation of nanoparticle uptake in hMSCs (**A**) and HeLa cells (**B**) using flow cytometry. Polystyrene nanoparticles on the surface of human serum at a concentration of 75 μg/mL incubated with cells for 24 h. (**C**) Corresponding Confocal microscopy images of living hMSCs. Plasma membrane stained with CellMaskTM Orange (pseudo red coloured), nucleus stained with Draq5 (blue), and nanoparticles labeled with Bodipy-1 (green). Scale bars = 75 μm. Adapted, with permission, from [62].

**Figure 8:**
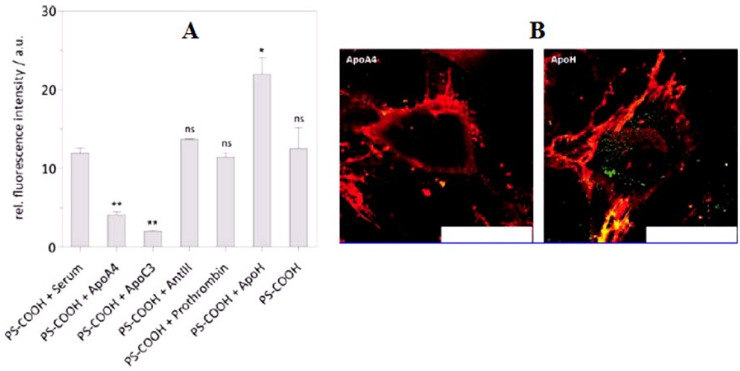
Impact of the single protein surfacing on nanoparticle cell uptake in hMSCs. (**A**) The polystyrene nanoparticle (carboxy functionalized, PSCOOH) was coated with ApoA4, ApoC3, AntIII, prothrombin, or ApoH (50 μg per 0.05 m^2^, 1 h, 37 °C), and incubated for 6 h in a serum-free medium. Error bars ± SEM estimated with two experiments in triplicate (*n* = 3). Level of significance (* *p* < 0.05; ** *p* < 0.01). (**B**) Accompanying confocal microscopy images of living hMSCs treated with ApoA4 or ApoH-coated carboxy functionalized polystyrene nanoparticle. Plasma membrane stained with cell mask orange (red) and nanoparticles labeled with Bodipy-1. Scale bars = 75 μm. Adapted, with permission, from [62].

The intelligent view on tissue selectivity and targeting wasdevelopeda few decades ago when the London based scientist P. Enrich coined the term “magic bullet” for drug delivery in a targeted manner and this still remains a useful key concept in drug targeting to various diseases [74]. In drug targeting, an important consideration is how the cargos accurately payoff their release to the corresponding target domain. NPs plausibly accumulate and access their target via two generalized targeting mechanisms: the passive and active approaches. The passive mode relies on the vascular permeability of the tumor microenvironment [75,76,77]. Due to the rapid division of tumor cells, the newly formed blood vessels may fail to become fully matured causing angiogenesis (abnormal blood vessel) leading to an uneven blood flow through this leaky vascular route and poor lymphatic drainage leads to an enhanced permeation and retention effect (EPR) [78,79]. The non-engineered NPs passively accumulate to the target via an EPR effect, a fundamental concept that stands for secure, safe, and effective therapeutic concentrations [80]. The clinical evidence has suggested that EPR-based therapy is inconsistent as it depends upon the variant tumor stage, the cancer type, the hypoxic condition, an enhanced tumor interstitial fluid pressure, and other factors [81]. Contrary to this, the active targeting approach is based on the receptor-mediated cellular entry of therapeutics. In this, the nanosystem is decorated with a specific ligand that enables the membrane surface receptor to bind with it. The surface-anchored ligand may be tuned to obtain the essential optimal conditions for the potential internalization of therapeutic moiety inside cells. The surface density, the accessibility and affinity of the ligand, and receptor expression level on the cell membrane are important consideration for specific targeting [82,83]. The efficacy of nanomedicine to the target solely depends on the how the targeting specimen retains the in vitro synthetic identity of the carrier system inside the biological system and enables them to release. However, the synthetic identity of NPs such as the particle size, surface topography, charge, polydispersity index and aggregation characteristics become altered when they are exposed to in vivo conditions because a large fraction of the serum protein is adsorbed by the development of primary and secondary bond with the NPs’ surface, thus impacting cell uptake. The uptake of the NPs is largely influenced by the adsorption of abundant corona on to the surface of NPs in the biological milieu. Mahmoudi et al. suggested that the fate of NPs in the biological milieu depends upon all NPs’ synthetic parameters that influence the nano–biointerface [61]. The critical understanding of PC of NPs depends ontheir interaction with the cell surface i.e., the nano–biointerface is essential for the safe and reliable design of high yield NPs for promising and effective therapy. The cellular uptake of corona particles has a mixed effect in biological machineries. It has been seen that bare NPs rapidly enter the cells free of serum protein compared to inaccessible corona particles. However, a protective layer formed by PC on the NPs’ surface is beneficial as it extenuates the acute toxicity level to the biological environment [84]. The molecular structure of PC in NPs is highly complex, and a single protein has significant role in the specific cell uptake of NPs. Considering the impact of corona NPs on cell uptake, an analysis was carried out by Ritz and associates. They differentially analyzed the corona layer composition on various functionalized polymeric carboxy (PS-COOH), amino (PSNH_2_), sulfonate (PS-SO_3_), or phosphonate (PS-PO_3_) NPs with the help of quantitative mass spectrometry (label-free). Further, they correlated the relative teemingness of known proteins in the PC with the positive or negative impact of NPs on cellular uptake in cancer and stem cells to discriminate the key corona component. Interestingly, the PS-COOH and PS-PO_3_ functionalized NPs were expeditiously taken up, whereas PS-NH_2_ and PS-SO_3_ had a lower uptake in both the cell lines, as shown in Figure 7A–C. Further, artificially anchored NPs with a single protein corona either by the apolipoproteins ApoA4 or ApoC3 demonstrated a significantly reduced cellular uptake as illustrated in Figure 8A, while pre-coating other corona proteins such as ApoH improved the cellular uptake, as shown in Figure 8B [62].

Further, to examine the impact of protein corona on the synthetic identity of lipid-based nanoparticles (LNP) in biological systems, Chen et al. detailed the analysis of PC NPs which revealed that there was a “missing connection” between the physicochemical properties of NPs and their biological identity. However, there is a limited understanding of the impact of protein corona on the targeted delivery of tumors. It was demonstrated that protein corona on the surface of NPs could be manipulated by the design and development of a formulation. The alteration in the composition of the lipid component in LNPs could potentially change the charge on the NPs’ surface and finally change the formed corona patterns in the serum of nude mice. Thus, lipid surface charge manipulation and a specific lipid switch on the NPs could alter the PC profile. In spite of this fact, negatively charged NPs had no profound effect on the formation of protein corona, while the institution of positively charged lipids into the nanosystem dramatically switched the pattern of PC from apolipoprotein to vitronectin-rich. The composition of this variant of protein corona had a great impact on cell transfection, biodistribution in vivo, and the efficient active delivery to tumor tissues. The corona rich with apolipoprotein demonstrated improved delivery to hepatocellular carcinoma mediated via the LDL receptor which was equated with vitronectin-rich NPs. Additionally, it was observed that a quantitative estimation of the PEG in the PEG-conjugated lipid chains in LNPs was the determinant factor for the successful transfection of siRNA for solid tumor therapy [85]. Deng and colleagues showed that negatively charged poly(acrylic acid)-coupled gold NPs adsorb and extend the fibrinogen from human serum, resulting in the release of inflammatory cytokines mediated via the positive interaction with the Mac-1 receptor [42]. For instance, an early report by Kreuter et al. addressed the fact that apolipoprotein E from serum adsorbed to polysorbate 80 NPs, which facilitated transport across the blood brain barrier [86]. Other studies have demonstrated that ionizable lipid nanoparticles bearing apolipoprotein-E (ApoE) ligand normally adsorb ApoE, resulting in a pronounced cell uptake into the liver cells mediated through several receptors withaffinity for the same [87]. Furthermore, DNA and 1,2-dioleoyl-3- trimethylammonium propane-bearing lipidic nanoparticles had a high level of vitronectin in human plasma that was readily taken up into tumor tissues bearing the overexpressed receptor vitronectin (integrin αvβ3) [88]. It has been shown that silica NPs interact with a specific receptor after the formation of PC in human serum with LDL, and it has also been shown that IgG prompted uptake interceded via an interaction with the LDL receptor and the Fc-gamma receptor I (FcγRI) [89,90].

## 10. Prediction of the NP–Cellular Interaction and Analysis

Erstwhile NPs in the biological system travel through the receptor actively targeting it in vivo with their biological identity as they lose their synthetic identity due to the surfacing of biomolecules in the serum. The functional characteristics of ligated NPs can be cloaked and as a consequence they may lose their targeting potential [15,91]. The protein surface on NPs can be made up of soft corona (the dynamic alteration persists for a short time) or hard corona (dynamic alteration persists for long time). The protein -bound layer (NP–protein interaction) affects the process of receptor identification, the NPs’ interaction with the cell receptor, the NP–cellular association, cell uptake, and the pharmacological and biological responses of NPs. It has been shown that the protein characteristics and their abundance over NPs surface encode valuable data to predict the fate of NPs in vivo [12].

A strategic approach to combat corona formation could be possible by maintaining the synthetic identity and targeting efficiency by means of introducing a stealthy covering using polyethylene glycol or by modifying the NPs’ surface with the formation of zwitterions. The surface adsorption of zwitterions on NPs is favorable for strong hydrophilic binding electrostatically and thus, reducing protein binding. The NP–protein interaction is a subject of great concern and more rigorous analysis is required to better understand the interaction of corona NPs with cell surface receptors. The prediction of nano–biointeraction is highly challenging due to the complexity in the biological environment, the cells, the tissues, and at the molecular level, and their correlation with the surface chemistry of NPs. The development of quantitative models is helpful in the prediction of the nano–biointeractions which may in turn translate into nanotechnology for such biological events. The analysis of corona particles relies on characterization techniques which involve a significant estimation of the physico-chemical properties of PC, and the application of mass spectrometry for elaborating the composition of PC.

Walkey and associates characterized the “fingerprint”of protein corona developed across a library of 105 functionalized gold nanoparticles. Using a computational approach, they developed a multivariate model using the protein corona fingerprint to anticipate cell association which is far better than the parameters which utilize the physico-chemical features viz. NPs size, surface charge, and aggregation state. The model entails several hyaluronan-binding proteins that act as mediators of nano–biointeraction. The developed framework for a database of PC fingerprints provides quantitative relationships to anticipate the biological responses of various types of NPs and to show the underlying mechanisms of nano–biointeractions [92].

## 11. Impact of Corona Particles on the Biodistribution and Pharmacokinetics of Drugs

NPs in the biological system wrapped by various biological proteins lead to the development of protein corona. The formation of a new biological identity of NPs interacting with cells and tissues in the living organism and their altered functions in the biological system leads to a decrease in the targeting efficiency because of the masking of the identity of the targeting ligand to the receptor of the cell membrane, or it may lead to the NPs being engulfed by the macrophagic system. While some PCs increase the targeting capability, this depends on the type and conformation of the protein. PC formations on the surface of the NPs modify the fate of nanomaterials regarding the biodistribution and circulation time in physiological fluid [65].

Tekie and associates assumed that without significantly modulating the physicochemical characteristics of nanomaterials pertaining to bio-interference, the effective therapeutic efficacy of the intended objective would be inconceivable. The team studied the generation and biodistribution of various chitosan (Ch)-based nanoparticles including Ch and carboxymethyl dextran (CMD)/thiolated dextran (TD) polyelectrolyte complexes (PECs) by applying the chromatography, mass spectroscopy, and CT scanning techniques. The changes in the surface adsorption of the serum protein in the culture medium after varying the pH and the consequent cell uptake were evaluated in vitro. It was shown that the developed PECs had a low concentration of PC normally enriched with apolipoproteins, trypsin, and haemoglobin. Moreover, the study reported the positive outcome of the PC layer on PECs which prompted biodistribution and a regressed uptake in the liver and achieved a desirable therapeutic effect.

In the biodistribution study, positron emission tomography-computed tomography (PET/CT) scan images of three animal groups apparently showed that the concentration of NPs unbounded 68 Ga in the kidneys and bladder which had filtered in this organ. Moreover, a significant concentration of NPs was found in the hearts of three animal groups due to the rapid uptake and accumulation of the NPs in the blood pool and tissue. The PET scan also determined that some fraction of NPs associated with protein corona composition enriched with lipoproteins was deposited into the heart tissue. Amazingly, the animal model showed a lower uptake of NPs in the lung site, which indicates the suitable size distribution of the NPs within the serum, the bigger size of particle that accumulated in the pulmonary tissue, and the poor uptake by the spleen. This study revealed that PC has an impact on the biodistribution and cell uptake process [65].

Xiao et al. investigated the fate of spherical nucleic acids (SNAs) in the biological milieu due to surface adsorption serum protein. With the help of proteomic analysis, it was shown that G-quadruplexes templated on the gold NPs’ surface, as the SNAs liaised the organization of PC rich in complement protein compared to the SNAs with poly-thymine (poly-T) DNA. The cell uptake studies demonstrated that the complement receptor on macrophage cells identified the PC of SNA, thus alleviating cell internalization due to the accumulation of G-rich SNAs in the liver and spleen compared to poly-thymine DNA SNAs in vivo. The findings support the claim that the rational design of a nucleic acid sequence can mediate nano–biointeraction and can modify their cell uptake and biodistribution characteristics, which are crucial parameters in the design SNA therapeutics [93].

## 12. Impact of the Disease State on Corona Particle Formation

The altered metabolic rate and/or lifestyle in the disease state of an individual influences their biological machinery such as the protein complex and the protein synthesis process, and their modification together on plasma proteomics brings changes in the formation of PC. For instance, the breakdown of low-density lipoproteins due to the glycation of proteins causes a considerable reduction in the serum albumin level [56]. Albumin is primarily synthesized by the liver and is required for the transport of vitamins and hormones in the body. Poor nutrition, infection, and/or liver disorder or other pathological conditions are indicative of changes in the albumin level. It is well understood that a small variation in the protein composition of plasma and serum in a biological medium brings substantial alterations in the corona formed on the surface of NPs. The term personalized corona is related to an individual’s health state that may vary from person to person in relation to age, race, habits, and geographical region. The same NPs incubated with plasma in the serum protein of a person with distinguished pathologies may produce PC of a different composition [94,95]. Smoking leads to a reduced level of 3-nitrotyrosine in the plasma protein. Exposure of nitrotyrosine to plasma protein reduces the level of serum albumin, fibrinogen, and surfactant proteins [96,97]. The auto-antibodies, protein biomarkers derived from an individual with cancer disease, have shown altered plasma proteome based on the analysis of the pattern of corona formation on the surface of NPs in the serum of such an individual, which may vary from the PC formed from the plasma of a healthy person. A study has shown that PCs developed around NPs (silica and polystyrene) in the plasma obtained from patients suffering from various ailments viz., including diabetes, and cancer, differed from the PCs in those with differing lifestyles including smoking, a high fat diet, and pregnancy conditions. PC patterns obtained on silver stained SDS-PAGE gels pointed out that the PCs formed in individuals with various diseases differed in both quantity and composition. Interestingly, the PCs developed in the plasma of patients with similar ailments and habits were almost similar. However, the pattern of the PC was generally not uniform in the plasma of a healthy person with the same gender and age [57]. Further, the influence on graphene oxide (GO) sheets was not unlike the biological responses when incubated in the plasma of individuals with different types of disease including cancer, diabetes, and pregnancy. The same GO sheets coated with a varying composition of corona exhibited a different cytotoxicity, cell uptake, nano-toxicity, and lipid peroxidation. The report from this study opens a door for the researcher to further explore the nano–biointeraction in relation to various disease states, and the corresponding formation of protein corona and will help in the rational design of nanocarriers for effective and safe drug delivery for application in living organisms [57].

## 13. Impact on the Pharmacological Activities through Altered Protein Conformation

Protein conformation is related to the dimensional structure or shape/size and orientation of the protein owing to the diverse transcription of amino acids or the peptide chain. The modification in the primary, secondary, or tertiary structure of the protein leads to a significant alteration in the pharmacological and biological activities because of dynamic modification of the exposed conditions in the biological milieu. The conformation changes in protein can take place once the NPs are exposed to the biological system owing to formation of the PC surrounding the NPs that can adapt to the shape of them. The minute change in the structural conformation in the protein may lead to a significant impact on the biological responses [98,99]. The presence of the surface charge strongly impacted the protein conformation of gold NPs with similar characteristics but the charge on the surface differed when a similar quantity of BSA was adsorbed. Moreover, positively charged NPs showed a greater and faster cell uptake due to the greater association with the cells compared to the negatively charged particles and the outcome suggested that the internal modifications in the structural geometry of BSA to bind with NPs could be due to the charged surface of NPs [66].

## 14. Impact on Cell Toxicity

The PC formation around the NPs in the blood stream may result in harmful effects to the living organism. They can trigger immune responses and affect the targeting capability of NPs and induce toxicity [100]. Borgognoni and coworkers investigated the corona-coated titanium NPs in response to BSA on macrophages. The elevated level of cytokines andIL-6 and IL-1β from human macrophages was observed when PC-coated NPs were exposed to macrophages in a concentration-dependent fashion [63]. The secondary modified proteins present in the PC layer enabled interactions with macrophages’ surface receptor and stimulated a protein signal resulting in the production of cytokine. This finding intimated that that the PC formation could lead to an easy identification of NPs by macrophages, thus promoting inflammation. C. Ge et al. investigated the impact of serum–protein interaction on single-wall carbon nanotubes (SWCNTs). The study revealed that the competitive binding of the serum protein with the SWCNT surface significantly altered their interaction with the target cells and thereby minimized their cytotoxicity and suggested a safe design of a carbon nanonmaterial with a pre-consideration of the cellular interaction [64].

## 15. Impact of Protein Corona on Immune Response

The immune system has the natural capability to recognize the self and foreign bodies due to the self-coordinated organization of cells. The immune system is considered to be an important barrier in drug delivery at the cellular level, especially at nano–biointerface. The dendritic cells (DCs) are the key component in the activation of immune cells. The receptors on these cells recognize many molecular patterns including molecular pattern associated with NPs and a pattern recognition receptor such as a Toll-like receptor. Upon the activation of DCs, an inflammatory response is triggered by the recruitment of T cells of adaptive immunity. If coated with hydrophobic bio-corona in a biological system, the NPs may be treated as danger signals by the immune system. The engineered NPs in the biological system layered with complex proteins can act as a nanomaterial-associated molecular pattern (NAMPs) which is recognized by the pattern recognition receptors (PRRs), and the Toll-like receptors of innate immunity. Their activation triggers inflammation and is an alarming condition for the adaptive immune system to recognize a danger signal [101]. When a complement protein forms protein corona with nanomaterials in the biological milieu, the activation of the complement process results in inflammation [102]. Therefore, the formation of PC may cause the immunogenicity of NPs and finally, immune toxicity.

The generation of a protein layer on the surface of NPs that leads to PC formation has critical influence on particle recognition by the immune system that elicits an immune response, in turn causing their rapid clearance by the phagocytic system in the organs involving the spleen, liver, and lungs. In relation to this, Giulimondi et al., prepared liposomes and investigated them in vivo to reveal the formation of a biomolecular layer on to the liposomal surface and the impact on their synthetic identity, and ultimately showed that the identity of liposomes in the biological milieu is associated with sequestration from the mononuclear immune system [103]. The prior technique of PEG grafting over the liposome surface has had limited success in preventing the opsonization process [104]. From this perspective, the pre-coating strategy on the surface of liposomes using in vitroprotein corona which simulates plasma protein could rigorously reduce the capture by mononuclear immune cells and improve circulation time in vivo. Additionally, the biomimetic and bioinspired approach which is “biologically inert” reducing nano–biointeraction and interaction with the immune cells of diverse groups of nanocarriers is an emerging trend in drug delivery and works by active engagement at the molecular level [105].

## 16. Lipid Corona

There is limited understanding of the presence of lipid molecules and their adsorption on nanocarriers in the scientific research. In the blood, a lipid associated with protein is known as a lipoprotein. It is complex of apolipoprotein, cholesterol, triglycerides, and phospholipids [106]. Apart from the transportation of lipoprotein in a biological fluid, it is also associated with several other biological processes including tissue repair, coagulation, and immunological responses.

The proteomic data have suggested that a significant amount of apolipoproteins are present in the protein corona of nanomaterials in vivo. Hellstrand and associates highlighted a report of lipid and plasma protein interaction with nanocarriers. They first pointed out the interaction of lipoprotein complexes with nanomaterials [107]. The surface-active agent present in the pulmonary route constituted the outset host defense in deeper part of lungs. The inhaled nanoparticle primarily formed a different corona protein due to the interaction with the lipid and the surfactant-rich pulmonary surface, but the protein corona formed here was different from the PC formed by plasma protein. Further, Raesch and coworkers presented the proteomic analysis of corona formation on NPs using a porcine surfactant as a prototype. The study analyzed the adsorbed biomolecules using LC-MS on to the surface of NPs with varying characteristics viz. lipid-NP, PEG, and PLGA NPs with incubation in a porcine surfactant. The quantitative measurements showed specific lipid compositions in coronas of all investigated NPs. The hydrophilic PEG NPs only showed minimal lipid concentrations, while other NPs indicated high lipid surface binding to nanomaterials. However, the corona formed on NPs was different from corona of plasma protein [108].

For the application of nanomaterials in clinics, the physico-chemical properties have to be critically evaluated in a biological medium and the estimation of the results of in vivo studies will greatly help in the clinical translation of nanomaterials. The mouse model is widely used in nanomedicine research to investigate the corona on NPs by incubation with mouse serum. Herein, the carboxylated polystyrene NPs of various sizes 26 nm, 80 nm, and 200 nm were estimated after incubation with mouse serum. The mass spectrometric analysis revealed alterations in particle size, zeta potential, and the composition of the protein. The size of the corona particle was quantified by estimating the level of triglycerides and the cholesterol presence across the NPs’ surface. Overall, the results showed that the lipid had a significant impact on the formation of bio-corona. Using mouse serum will be helpful for preclinical studies and the clinical translation of NPs [109].

## 17. Conclusions and Future Perspectives

Despite the growing utility of NPs in the pharmaceutical and cosmetics industries, nanodrug delivery, theranostics, and bioelectronics, their application in bio–nanomedicine remains circumscribed. Even given the intensive characterization of NPs in vitro and the manipulation of their surface chemistry through standardized techniques, their synthetic identity remains doubtful in the biological milieu due to the complex nature of the biological medium and PC formation. The evolution of PC on the surface of NPs in living organisms gives them a biological identity that puts a question mark on the potential of nanomedicine delivering therapeutics to the target. PCs are tightly bound and have complex structures, and they may determine the fate of NPs as biological responses in living organisms. The impact of PC is not limited to the NPs’ surface in physiological media but their influence can be sees at the cell, tissue, and organ/system levels, and in particular, in drug delivery, kinetics, targeting, cell uptake, and altered therapeutic efficacies in the biological system. The receptor-based targeting approach is useful in overcoming the biological barriers to reach out to the cells for the active targeting of therapeutics. However, an active target particle covered with corona in vivo is unable to decipher the precise receptor on the cell membrane and fails to deliver the drug in target. To date, the underlying mechanisms and the understanding of nano–biointeraction is limited, and broader study is required to explore them in the scientific domain. Further, taking into account the consideration of PC evolution in biological media, NPs could be synthesized in such a way that their surface would have limited access by PC and a low impact on the biological responses. To explore this, the knowledge based on recent approaches to the flow dynamics of biological fluids should be taken into consideration to give an insight into the precise evaluation of the NP–protein interactions. Further, the influence of the shear stress on PC formation on every nanomaterials needs to be critically investigated. The PC fingerprint is helpful in predicting the interaction of corona-layered NPs with targets. The development of a predictive model based on epitope-mapping, a tool that accurately draws the functional indication of a biomolecular motif at the nano–biointerface should be strategically established. To this end, the formation of PC on the surface of NPs in vivo and their recovery needs to be thoroughly characterized, investigated, and reproduced clearly in order to evaluate their biological interaction. We hope that future developments in this domain will provide more insight and bring out favorable opportunities to accelerate the translation of nanomedicines into clinics.

## Figures and Tables

**Table 1:** Effect of the physico-chemical characteristics of nanomaterials in PC formation in the biological milieu.

Factors	Impact on the Fate of NPs via Interaction with PC in a Biological Medium	Ref.
1. Physico-chemical characteristics of NPs	1. Small size particles have a large surface curvature resulting in a poor influence on the protein’s conformation. 2. Bigger particles have a large surface area for individual protein interaction. 3. If the surface area is large, low affinity proteins may bind and stabilize the interaction in the aggregates of NPs. 4. Particle shape alters the mass/surface area ratio; spherical particles minimize the interaction. 5. Slightly negatively charged proteins appear to have lower interactions with proteins.	[29,34,35]
1.1. Surface charge	1. Obtusely charged NPs incline towards higher and denser PCs. 2. Positively charged NPs rapidly and strongly bind with proteins with an isoelectric point of less than 5.5. 3. Highly negatively charged NPs interact mostly with proteins with an iso-electric point greater than 5.5. 4. Less negatively charged NPs have poor interactions with proteins.	[37,38]
2. Experimental and environmental factors affecting PC formation		
2.1. Incubation medium	The concentration of proteins and the composition of the biological fluid (plasma, serum, interstitial fluid) have an effect upon PC formation. The animal species such as rats, mice, bovine, or human have impact on PC formation. The samples obtained from humans of varying ages, sex, diets, states of health and inter-individual variabilities have an influence on PC.	[6,50]
2.2. Flow dynamics	The dynamic nature of blood flow in the human body cause stress for NPs, a source of PC adsorption. The un-PEGylated NPs show a higher concentration of PC and evolve into apolipoproteins APOA-II under dynamic conditions, while under static conditions, acute phase proteins and alpha-1-antitrypsin were recorded.	[51,52,53]
2.3. Temperature, time of incubation, and pH	Increasing the incubation temperature of the serum from 25 to 70 °C with NPs leads to denatured PC covers. A report showed that the PEGylated gold NPs of size 30 nm incubated in plasma at room temperature and 37 °C showed that the concentration of proteins recovered decreased with an increase in time from 5 to 60 min. The fluorescence correlation spectroscopy established that the binding feature of BSA to QDs changed pH from 6 to 9. At a lower pH, the binding affinity was lower due to a repulsive force. At a higher pH, higher binding to QDs was observed because of conformation alteration in the protein structure.	[6,54,55]
3. Disease state	Individual disease states that change the metabolic rate or lifestyles have an influence on the protein complex and plasma proteomics that bring achange in the PC formation. For instance, protein glycation causes a considerable reduction in the serum albumin level. A study on the SDS-PAGE gels on silica and polystyrene revealed that the PCs differ in both quantity and composition invaried disease states.	[56,57]
4. Drug release	1. The PC layers around the NPs’ surface reduce the effective burst release profile of the commercially available product Abraxane^®^ 2. Camptothecin release from silica NPs showed a slower release due to protein corona. 3. Sebak et al. compared the PC concentration to bare polymeric PLGA-NPs and peptide ligated hybrid NPs (cRGDyk peptide) when incubated in plasma. They established that the in vitro drug release from the NPs largely depended on the PC composition as well as the concentration of serum proteins in the medium. A higher release rate was recorded for peptide-conjugated NPs and a reduced drug release rate was recorded for bare PLGA-NPs.	[58,59,60]
5. Influence on drug targeting and cellular uptake	The understanding of PC of NPs and their interaction with the cell surface i.e.,the nano–biointerface is essential for promising and effective therapy. The cellular uptake of corona particles has mixed effects in biological machineries. Some studies have reported that a protective layer on the NPs’ surface extenuates the acute toxicity level of the biological environment. Artificially anchoring NPs with a single corona by apolipoproteins ApoA4 or ApoC3 led to a significant reduction in cellular uptake, while pre-coating with the corona protein ApoH improved the cellular uptake.	[61,62]
6. Impact of PC on cell toxicity	Apart from affecting drug delivery, targeting, and cellular internalization, PC also impacts nano-toxicity and triggers disease pathophysiology. The PC (transferrin, globulin, BSA, and BGF)-layered SWCNT expressed a comparatively lower cytotoxicity than bare SWCNTs. The PC layer formed on titanium dioxide NPs formed in human macrophages resulted in an enhanced secretion of inflammatory cytokines, viz., IL-1β, IL-6, and IL-10 from macrophages that rely on the concentration of NPs.	[63,64]
7. Impact of corona particles on the biodistribution and pharmacokinetics of drugs	The biological identity of NPs that differ from the in vitro design interact with living tissues and their functions in the biological system alter, resulting in a decrease in the targeting efficiency due to the PC covering. They may be taken up by RES. PC sometimes aid in the increase of the the targeting capability but this depends on the type and conformation of the protein. PC formations on the NPs’ surface modify the fate of nanomaterials in relation to biodistribution and the circulation time in physiological fluid.	[65]
8. Impact on pharmacological activities	The alteration in the primary/secondary/tertiary structure of the protein leads to significant alterations in the pharmacological and biological activities.	[66]

**Table 2:** Some examples of nanomaterials integrating with proteins forming corona in blood plasma/serum.

Nanomaterials	Incubation Medium	Protein Corona Compositions	Inference	Ref.
Iron oxideNPs/SPION	FBS	Anti-thrombin, α-antiproteinase, and serotransferrin.	Polyvinyl alcohol (PVA)-coated SPIONs with (−) and (+) surface charge had a higher adsorption rate in serum proteins than the dextran-coated SPIONs that led to a higher circulation time in blood in the case of PVA-coated NPs compared to the dextran-coated SPIONs.	[21]
Magnetic NPs	Human blood serum and human lymph serum	Serum albumin, Apolipoprotein A-I, Prothrombin, Plasminogen, Complement protein, Apolipoprotein B-100, Apolipoprotein E, Antithrombin-III, Vitronectin, and Kininogen-1	Hard protein corona (HPCs) received by two isolation methods were entirely different by upto 50%, which suggested that only these proteins that were found in the HPCs fromboth magnetic separation and multistep centrifugation methods were real HPCs.	[22]
Artificial viral NPs with AuNPs	Blood serum	Reported presence of hard and soft corona on nanoparticles. Despite corona evolution over NPs, GM3 enclosed in the AVN membrane remained approachable to CD169 receptor binding.	The bigger particles with low DOPS % showed a higher stability in serum plasma. As a result, a increased layering of PC led to a lowering in the targeting of GM3 for CD169. Further, study is required to give insight into the formation of PC with regard to AVN in vitro, although this extends key points of relevance to PC layering on NP size and fate in the biological environment	[29]
AuNPs	-	Serum albumin, Alpha-2-Macroglobulin, Apolipoprotein A-I, Apolipoprotein E, Complement factor H, Plasminogen, Ig mu chain C region, Protein Ighv7–1.	The results indicated from the gel electrophoresis and mass spectrometry analysis that the development of the complex with protein coronas, took place within 10 min of injection.	[30]
Gold nanostructures (spheres, rods, stars, and cages)	70% human serum (diluted with PBS) for 2 h	The 15 most abundant proteins were associated AuNPs. Some of them were Serum albumin, Apolipoprotein E, Coagulation factor XII, Apolipoprotein A-I and A-II, Kininogen-1, Gelsolin, Vitronectin, Histidine-rich glycoprotein.	The cage-like structure of AuNPs indicated the lowest adsorbed corona proteins. The results revealed that nano-cages could improve the compatibility with the biological medium compared with other shapes due to the high area of curvature and the heavy ligation over flat surfaces that opposes opsonization and the rapid clearance via the immune system.	[32]
Nanoparticles (silica, polystyrene, and carboxyl-modified polystyrene particles)	Human plasma; plasma with cytosolic fluid	Tubulin alpha-1, Alpha-enolase, Nucleophosmin, Protein S100-A9, 60S ribosomal protein L14, PEST proteolytic signal-containing nuclear protein, Triosephosphate isomerase, Protein S100-A9.	The results have shown that abundant protein corona could evolve in the II^nd^ biological solution, but the last protein left a “fingerprint” of its history. This is important to map the evolution and understand how the pathway was generated for adsorption to the nanoparticles, and eventually to predict the fate and behavior of the nanoparticles.	[35]
PSCOOH, PSOSO_3_H, and silica particles (SiO_2_)	Blood plasma	Hard and soft corona particles on the nanoparticle surface altered their surface chemistry.	Formation of hard or soft corona protein assembly and their longevity depends upon the nanomaterial type. The blood plasma-derived protein coronas have a long life. Rather than appearing over the surface of the nanomaterial, this is actually what the cell sees.	[19]
CS NPs	FBS, biological buffer, and serum	Protein coronas of different compositions	Protein corona adsorption on the HA-chitosan nanoparticle influenced the interaction with the HA-receptor i.e., CDD4 mediated cellular uptake.	[37]
Colloidal silica nanoparticles	FBS in Phosphate Buffer Saline (PBS)	Protein corona of varying molecular weight ranges (MW< 17 kDa to >135 kDa) were accessed on the silica particle according to the protein band intensity.	The colloidal destability of the nanoparticles was overcome by adding depletant polymers, Pluronic-F127 and PEG, of different molecular weights. The interaction between the polymer and the nanoparticle had a minimal impact on protein access by the nanoparticle surface upon incubation with serum. The serum protein had a significant effect on the corona profile compared to other polymers.	[9]
AgNPs	Model protein environments for the self-evolution of corona	Model protein BSA	These polymers, polyethyleneimine (PEI), polyvinylpyrrolidone (PVP), and poly(2-vinyl pyridine)-b-poly(ethylene oxide) (PEO-b-P2VP) were applied as stabilizing agents. The PEO-b-P2VP and PVP-stabilized nanoparticles were reported to be inert to the protein’s adsorption. The PEI-stabilized AgNPs had substantial interactions with BSA.	[10]
